# Medical nutrition therapy in incretin-treated obesity-related heart failure with preserved ejection fraction

**DOI:** 10.3389/fnut.2026.1914155

**Published:** 2026-08-14

**Authors:** Qiuli Ming, Ze Li

**Affiliations:** 1 The Third Affiliated Hospital of Henan Medical University, Xinxiang, China; 2 The First Affiliated Hospital and College of Clinical Medicine, Henan University of Science and Technology, Luoyang, China

**Keywords:** body composition, HFpEF, incretin-based therapy, medical nutrition therapy, muscle function, obesity

## Abstract

Glucagon-like peptide-1 receptor agonists and dual agonists of the glucose-dependent insulinotropic polypeptide and glucagon-like peptide-1 receptors, exemplified by semaglutide and tirzepatide, respectively, have reshaped care for obesity-related heart failure with preserved ejection fraction (HFpEF). Their benefits extend beyond weight reduction to symptoms and physical function and, for tirzepatide, a lower risk of worsening heart failure. Yet kilograms lost do not reveal whether treatment preferentially reduces harmful adiposity, preserves muscle function, or maintains nutritional adequacy. Against this background, we examine how medical nutrition therapy (MNT) can be integrated with incretin-based therapy to support high-quality weight loss—a working construct used here to denote loss of excess adiposity without avoidable compromise of nutritional status or functional reserve. The discussion centers on three linked questions: how appetite suppression and gastrointestinal intolerance alter nutritional risk; how body composition relates to muscle and patient-centered outcomes; and how nutrition care should evolve from treatment initiation through plateau, maintenance, and discontinuation. Available evidence supports early nutrition assessment, attention to protein and overall diet quality, and coordinated resistance and aerobic exercise, while showing that body composition cannot replace direct assessment of strength, walking capacity, symptoms, and health status. We therefore outline a phase-based MNT pathway for obesity-related HFpEF, while emphasizing that no randomized trial has tested a protocolized nutrition program alongside incretin-based therapy in this population. The next advance should be judged not only by the amount of weight lost, but by whether that loss is safe, function-preserving, durable, and meaningful to patients.

## 1 Introduction

### 1.1 Clinical burden of heart failure with preserved ejection fraction and the obesity phenotype

Heart failure (HF) with preserved ejection fraction (HFpEF) encompasses a heterogeneous clinical syndrome in which congestion, impaired exercise reserve, systemic inflammation, renal dysfunction, and metabolic disturbance jointly drive morbidity despite preserved left ventricular ejection fraction (1). Accordingly, contemporary pathways first establish the HFpEF phenotype, exclude mimics, stabilize volume status, and address hypertension, diabetes, atrial fibrillation, chronic kidney disease (CKD), obstructive sleep apnea (OSA), and obesity before assigning dyspnea or edema to a single cardiac mechanism (2). Obesity now occupies a central position within this diagnostic landscape, amplifying symptoms, physical limitation, volume stress, and cardiometabolic multimorbidity (3). Its management in HF therefore extends beyond a target body weight to encompass adiposity, functional capacity, cardiorenal risk, selection of obesity therapy, and longitudinal surveillance within the HF pathway (4).

Recent obesity frameworks formalize the separation between excess adiposity as a disease state and body mass index (BMI) as a screening metric, particularly when organ dysfunction, impaired mobility, or limitations in daily life are present (5). Contemporary staging systems likewise integrate anthropometry and fat distribution with medical and functional complications and the need for treatment intensification (6). In HFpEF, this distinction is clinically salient: Prospective Comparison of Angiotensin Receptor–Neprilysin Inhibitor With Angiotensin Receptor Blocker Global Outcomes in Heart Failure With Preserved Ejection Fraction (PARAGON-HF) documented near-universal central adiposity by waist-to-height ratio, with closer alignment to adverse HF risk than BMI categories (7). Participant-level analyses of large trials of heart failure with mildly reduced or preserved ejection fraction similarly show that waist circumference and waist-to-height ratio refine risk beyond BMI, supporting an obesity-related HFpEF phenotype assessed through adiposity, congestion, symptoms, exercise capacity, and functional reserve in combination (8).

### 1.2 Why incretin-based therapies have changed the treatment context

Weight management has become clinically relevant in HF as obesity is increasingly treated as a modifiable driver of symptoms, functional limitation, and cardiometabolic risk (9). Incretin-based therapies have broadened this therapeutic context from glucose lowering to a cardiometabolic platform spanning obesity, cardiovascular risk, kidney disease, and other obesity-related conditions (10). In Semaglutide Effects on Cardiovascular Outcomes in People With Overweight or Obesity (SELECT), semaglutide 2.4 mg lowered the incidence of major adverse cardiovascular events among adults with overweight or obesity, established cardiovascular disease, and no diabetes, placing cardiovascular risk modification alongside weight reduction (11). Long-term tirzepatide treatment produced substantial, durable pharmacologic weight loss and reduced progression from prediabetes to type 2 diabetes (T2D) in people with obesity (12). A prespecified SELECT analysis also associated semaglutide with better cardiovascular outcomes in participants with prevalent HF; the broader HF population, however, does not provide direct evidence for obesity-related HFpEF alone (13).

Semaglutide Treatment Effect in People With Obesity and Heart Failure With Preserved Ejection Fraction (STEP-HFpEF) provided phenotype-specific evidence, with semaglutide improving HF symptoms, physical limitations, exercise function, and body weight in patients with HFpEF and obesity (14). STEP-HFpEF DM extended these findings to obesity-related HFpEF with T2D, confirming relevance in a metabolically complex subgroup (15). A Study of Tirzepatide in Participants With Heart Failure With Preserved Ejection Fraction and Obesity (SUMMIT) further raised expectations: tirzepatide reduced the composite of cardiovascular death or worsening HF while improving health status, body weight, and walking capacity in HFpEF with obesity (16). The diabetes-stratified analysis sharpened an important qualification—patients with T2D lost less weight yet derived similar HF benefit, so body weight change alone cannot capture treatment value (17). Collectively, these trials open a therapeutic window in obesity-related HFpEF while redirecting attention to intake adequacy, body composition, durability of symptom relief, and long-term support, thereby bringing medical nutrition therapy (MNT) into the core treatment pathway (18). The key clinical evidence and its nutrition-relevant implications are summarized in Table 1.

**Table 1:** Key clinical evidence of incretin-based therapies in obesity-related HFpEF and their nutrition-relevant implications.

Study design/evidence source	Population/context	Incretin-based therapy	Key clinical signal	Nutrition-relevant implication	References
Phase 3 RCT; STEP-HFpEF	Obesity-related HFpEF without T2D; *n* = 529	Semaglutide 2.4 mg vs. placebo; 52 weeks	Weight −13.3 vs. −2.6%; KCCQ-CSS +7.8; 6MWD +20.3 m	No dietary, protein, micronutrient, or lean mass phenotyping	(14)
Phase 3 RCT; STEP-HFpEF DM	Obesity-related HFpEF with T2D; *n* = 616	Semaglutide 2.4 mg vs. placebo; 52 weeks	Weight −9.8 vs. −3.4%; KCCQ-CSS +7.3; 6MWD +14.3 m	T2D-specific glycemic, renal, and intake monitoring	(15)
Pooled individual-level RCT analysis	STEP-HFpEF and STEP-HFpEF DM; *n* = 1,145	Semaglutide 2.4 mg vs. placebo; median 401 days	Weight ETD −8.4%; KCCQ-CSS +7.5; 6MWD +17.1 m; CRP and NT-proBNP reduced	Strong efficacy signal; structured nutrition endpoints absent	(19)
Prespecified STEP-HFpEF analysis	HFpEF with obesity across BMI classes	Semaglutide; achieved weight loss strata; 52 weeks	Benefit consistent across BMI classes; larger weight loss linked to larger KCCQ and 6MWD gains	Weight loss quality unresolved; fat vs. lean mass loss unmeasured	(31)
Pooled prespecified diuretic use analysis	STEP-HFpEF program by diuretic category	Semaglutide 2.4 mg vs. placebo; 52 weeks	Weight benefit consistent; greater KCCQ benefit in loop-diuretic users; loop dose decreased	Fluid balance, renal monitoring, and diuretic adjustment	(143)
Phase 3 RCT; SUMMIT	HFpEF, EF ≥50%, BMI ≥30 kg/m^2^; *n* = 731	Tirzepatide up to 15 mg vs. placebo; median 104 weeks	Weight −13.9 vs. −2.2%; cardiovascular death or worsening HF; HR 0.62; KCCQ-CSS +6.9; 6MWD +18.3 m	Event-level efficacy with GI discontinuation signal; intake monitoring needed	(16)
SUMMIT clinical-trajectory analysis	HFpEF with obesity	Tirzepatide vs. placebo; median 104 weeks	KCCQ, 6MWD, EQ-5D-5L, PGIS, NYHA functional class, and medication burden improved	Function, HRQoL, and medication burden as nutrition pathway endpoints	(57)
SUMMIT mechanistic secondary analysis	HFpEF with obesity	Tirzepatide vs. placebo; 52 weeks	Estimated blood volume −0.58 L; CRP −37.2%; volume reduction correlated with KCCQ and 6MWD	Fluid, blood pressure, renal, and inflammatory monitoring during MNT	(144)
SUMMIT CMR substudy	Obesity-related HFpEF with analyzable CMR; *n* = 106	Tirzepatide vs. placebo; 52 weeks	Paracardiac adipose tissue −45 mL; LV mass −11 g	Adipose distribution as a target beyond body weight	(33)
SUMMIT analysis of BMI, central adiposity, and weight loss	HFpEF with obesity across BMI and central adiposity strata	Tirzepatide vs. placebo; 52 weeks	Greater weight and waist reduction linked to larger KCCQ and 6MWD gains	Waist and central adiposity tracking in nutrition follow-up	(32)

6MWD, 6-min walk distance; BMI, body mass index; CMR, cardiac magnetic resonance; CRP, C-reactive protein; DM, diabetes mellitus; EF, ejection fraction; EQ-5D-5L, EuroQol 5-Dimension 5-Level questionnaire; ETD, estimated treatment difference; GI, gastrointestinal; HF, heart failure; HFpEF, heart failure with preserved ejection fraction; HR, hazard ratio; HRQoL, health-related quality of life; KCCQ, Kansas City Cardiomyopathy Questionnaire; KCCQ-CSS, Kansas City Cardiomyopathy Questionnaire Clinical Summary Score; LV, left ventricular; MNT, medical nutrition therapy; NT-proBNP, N-terminal pro-B-type natriuretic peptide; NYHA, New York Heart Association; PGIS, Patient Global Impression of Severity; RCT, randomized controlled trial; T2D, type 2 diabetes.

### 1.3 Why nutrition is not an adjunct but a determinant of efficacy and safety

Pooled data from the STEP-HFpEF program show that semaglutide improves symptoms, physical limitations, exercise function, and body weight across obesity-related HFpEF with and without T2D; the relevant question now is how to make pharmacologic weight loss durable, function-preserving, and clinically worthwhile (19). Incretin-based therapy simultaneously changes appetite, food intake, gastrointestinal (GI) tolerance, and adherence, creating foreseeable needs for baseline nutrition assessment, prevention of nutrient deficiencies, and protection of muscle and bone (20). Proposed strategies call for early attention to muscle-related tissue loss during active weight reduction, supported by protein quality, targeted supplementation when indicated, physical activity, and body composition assessment (21). Even these safeguards require contextual interpretation, since neither scale weight nor a single body composition measure can define treatment quality or functional status (22).

Skeletal muscle warrants particular attention: it sustains mobility, glucose disposal, amino acid reserve, and metabolic resilience, all of which may already be compromised in obesity-related HFpEF (23). With the magnitude of weight loss now achievable during incretin-based therapy, body composition, protein adequacy, and exposure to resistance training become measurable components of treatment quality (24). In the STEP-HFpEF program, semaglutide reduced body weight across frailty strata and improved HF-related symptoms, highlighting the need to interpret benefit and nutritional risk in light of baseline functional vulnerability (25). Exercise-function analyses reached a similar conclusion, as symptom and physical-limitation improvements were accompanied by gains in 6-min walk distance (6MWD) across the full spectrum of baseline exercise capacity (26). The practical challenge is to coordinate pharmacologic weight loss with nutrition, muscle preservation, and direct functional assessment, allowing reductions in adiposity to be judged alongside meaningful improvements in symptoms and performance (27).

### 1.4 Literature search and evidence selection

PubMed and the Web of Science Core Collection were searched for English-language publications through June 15, 2026, without a lower date limit. Search terms combined HFpEF and obesity with incretin-based therapy, medical nutrition therapy, body composition, skeletal muscle, exercise, gastrointestinal adverse events, treatment discontinuation, and weight regain. HFpEF-specific randomized trials and prespecified analyses were prioritized. Relevant guidance documents, systematic reviews, and human studies in obesity or heart failure were used when direct HFpEF evidence was limited, and experimental studies were included when they informed skeletal-muscle mechanisms. Reference lists of key articles were screened, and the evidence was synthesized narratively without meta-analysis or formal risk-of-bias assessment.

## 2 From weight loss to high-quality weight loss

### 2.1 The concept of weight loss quality

In obesity-related HFpEF, high-quality weight loss denotes preferential reduction of excess adiposity while metabolic and physical function are preserved (28). Contemporary obesity care already evaluates treatment through changes in obesity-related disease, health status, everyday function, and risk burden, with kilograms lost serving as only one measure (29). This distinction is especially important in anti-obesity medication (AOM) trials, in which body weight and BMI cannot identify whether change arises from visceral or subcutaneous fat, water, organ mass, lean soft tissue, or skeletal muscle, or establish whether a patient is stronger, less congested, or more active (30). In STEP-HFpEF, semaglutide improved symptoms, physical limitations, exercise function, and body weight across obesity classes, and larger weight reductions accompanied greater clinical gains; weight remains informative, but cannot serve as a complete index of treatment quality (31).

SUMMIT adds a complementary perspective, as baseline BMI, central adiposity, and on-treatment weight loss were associated with heterogeneity in tirzepatide response, making the adiposity phenotype central to interpretation (32). Cardiac magnetic resonance findings from SUMMIT broaden this view beyond anthropometry: reductions in left ventricular mass and paracardiac adipose tissue connect regional adiposity with cardiac structure (33). A complication-oriented pharmacologic approach supports this reading by matching obesity treatment to the diseases and functional consequences under treatment, with efficacy judged on more than the amount of weight lost (34). For patients with HFpEF, beyond-the-scale targets include symptoms, physical limitations, walking capacity, health-related quality of life (HRQoL), body composition, and durability—measures that more closely reflect clinical state than percentage weight loss (35). Precision in language is essential when body weight, fat mass, lean mass, fat-free mass, skeletal muscle mass, myosteatosis, and function denote related yet noninterchangeable domains of weight-loss quality (36).

### 2.2 Distinguishing lean mass, fat-free mass, skeletal muscle, and myosteatosis

Interpreting weight-loss quality begins with terminology that distinguishes the major levels of body composition, as a change in body weight may reflect fat, water, organ mass, lean soft tissue, or skeletal muscle (37). At the molecular level, fat mass and fat-free mass constitute a two-compartment model, whereas lean body mass includes small lipid compartments and is often used imprecisely as a synonym for fat-free mass (38). Measurement introduces further ambiguity across dual-energy X-ray absorptiometry (DXA), bioelectrical impedance analysis (BIA), computed tomography, and magnetic resonance imaging (MRI), which estimate different compartments, incorporate distinct assumptions about water and tissue, and variously report fat, lean soft tissue, muscle volume, or muscle quality (39). Skeletal muscle mass is a more specific tissue–organ compartment than total lean mass; imaging and appendicular lean indices can approximate it, but body weight and whole-body lean mass cannot identify it directly (40). Serial BIA and DXA can be clinically useful provided their outputs are read within method-specific assumptions, calibration procedures, hydration sensitivity, and appropriate reference ranges, without treating the methods as interchangeable (41).

Muscle quality must also be considered separately, as it describes the strength or physical performance generated by a given quantity of muscle and may deteriorate despite relatively preserved mass (42). Myosteatosis represents one structural dimension of impaired quality, reflecting lipid accumulation within or between skeletal muscle fibers that may promote inflammation, insulin resistance, mitochondrial stress, and inefficient contraction (43). In the SURMOUNT-1 DXA substudy, tirzepatide reduced body weight, fat mass, and lean mass, underscoring the need to report the composition of pharmacologic weight loss (44). SEMALEAN complements these data by assessing fat mass, lean mass, and muscle function together during semaglutide treatment, keeping body composition distinct from functional testing (45). During intentional weight loss, a decline in lean soft tissue or measured muscle indices should be interpreted against strength, gait, physical performance, symptoms, and baseline vulnerability before it is labeled clinically harmful muscle loss (46). In obesity-related HFpEF, this distinction has direct clinical consequences because fat loss, lean mass, skeletal muscle mass, myosteatosis, and function are separate endpoints that together determine whether functional reserve is maintained (47).

### 2.3 Why functional reserve matters more in HFpEF

Preserved ejection fraction can obscure the nature of HFpEF, a syndrome defined clinically by exercise intolerance, congestion, impaired quality of life, and reduced physiological reserve (48). Exercise intolerance emerges from interacting deficits in cardiac filling and output reserve, pulmonary and vascular coupling, chronotropic response, peripheral oxygen delivery, and skeletal muscle oxygen use (49). Noncardiac comorbidity compounds these constraints, with greater burden associated with poorer exercise capacity and functional status in chronic HF (50). Deep phenotyping points in the same direction: patients who express multiple HFpEF phenogroup traits have lower exercise capacity, more adverse hemodynamic responses, and higher clinical risk, making a uniform weight-loss target an inadequate measure of benefit (51). Regional adiposity may further limit reserve in obesity-related HFpEF, given the association between excess pericardial fat and adverse cardiomechanical interaction (52).

**Figure 1:**
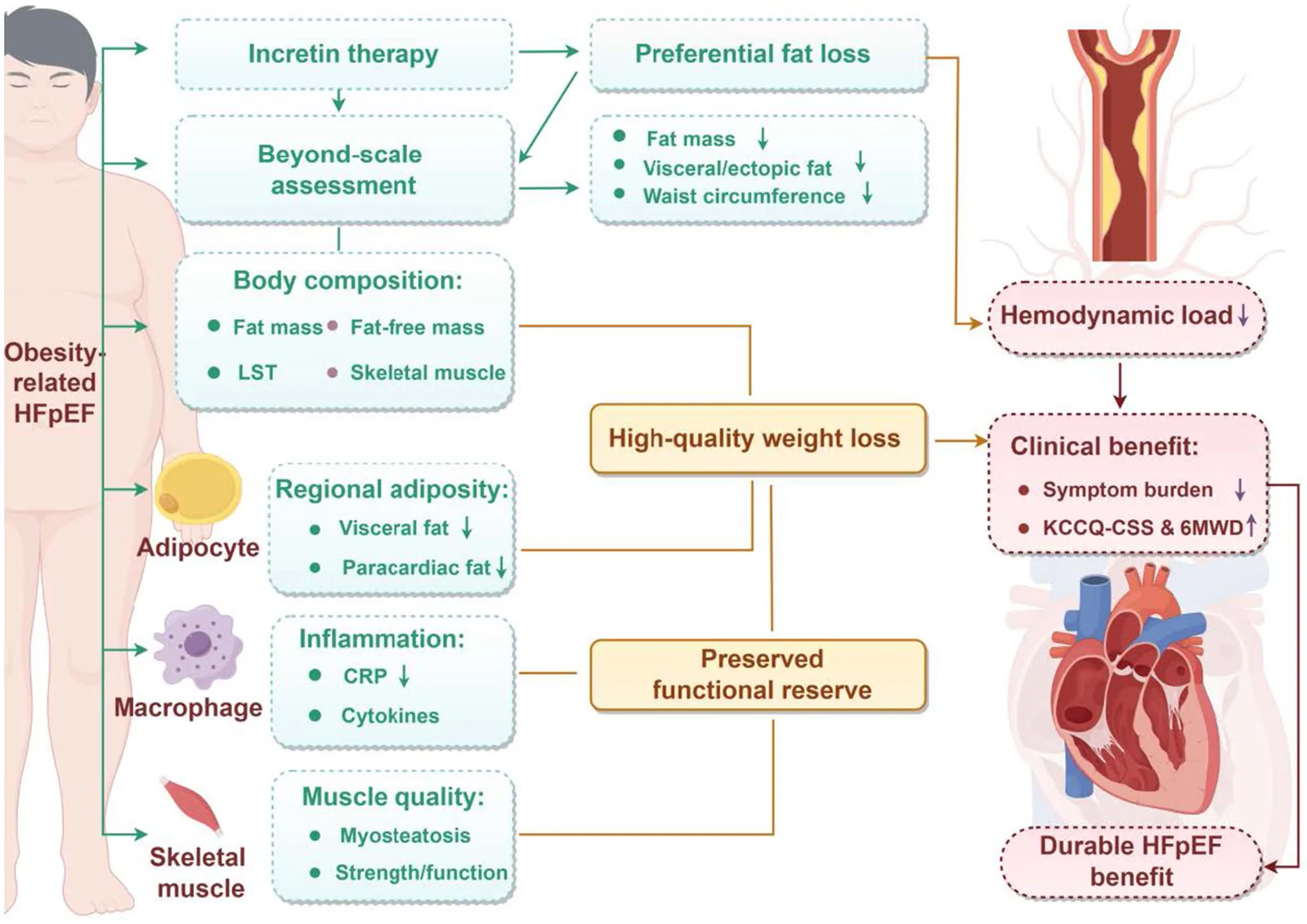
Beyond-the-scale framework for high-quality weight loss in obesity-related HFpEF during incretin-based therapy. High-quality weight loss is conceptualized as preferential reduction in excess adiposity with preservation of metabolic and physical function rather than reduction in scale weight alone. Incretin-based therapy may promote preferential fat loss, but fat-loss quality requires beyond-the-scale assessment of fat mass, visceral or ectopic fat, waist circumference, body composition, regional adiposity, inflammation, muscle quality, and functional reserve. Fat-free mass, lean soft tissue, skeletal muscle, myosteatosis, and strength and function represent related but noninterchangeable domains; therefore, body-composition findings should be interpreted together with direct functional measures. Preferential fat loss may reduce hemodynamic load, while high-quality weight loss and preserved functional reserve are linked to clinical benefit, including lower symptom burden and improvements in KCCQ-CSS and 6MWD. Durable HFpEF benefit represents the longitudinal clinical goal. Arrows indicate conceptual treatment, assessment, or outcome pathways; the arrow from preferential fat loss to beyond-the-scale assessment indicates that preferential fat loss should be confirmed and monitored through beyond-the-scale measures, not that assessment is a biological consequence of fat loss. Plain connector lines indicate assessment or definitional relationships rather than proven causal mediation. The figure was created using Figdraw. 6MWD, 6-min walk distance; CRP, C-reactive protein; HFpEF, heart failure with preserved ejection fraction; KCCQ-CSS, Kansas City Cardiomyopathy Questionnaire Clinical Summary Score; LST, lean soft tissue.

Peripheral dysfunction gives weight-loss quality an immediate nutrition–muscle dimension, as some patients with HFpEF exhibit impaired skeletal muscle oxygen transport and utilization during exercise (53). Biopsy studies also identify mitochondrial respiratory abnormalities that track with exercise intolerance, linking muscle metabolic capacity to symptoms and physical performance (54). Among older patients with HFpEF, exercise intolerance and outcomes may correspond more closely to impaired muscle metabolic quality than to age-related differences in muscle quantity (55). At the bedside, patient-reported frailty captures part of this vulnerability through its associations with Kansas City Cardiomyopathy Questionnaire (KCCQ) physical limitation and 6MWD and its prognostic value for subsequent functional status (56). The SUMMIT clinical-trajectory analysis extends the argument beyond body weight, documenting improvements in health status, quality of life, New York Heart Association (NYHA) functional class, walking distance, global symptom impression, and medication burden with tirzepatide (57). Nutritional status belongs in the same reserve-based assessment: transferrin saturation, more than ferritin, has been associated with metabolic and hemodynamic exercise responses in HFpEF, suggesting that nutrient-related deficits may influence the very outcomes used to judge treatment quality (58) (Figure 1).

## 3 Nutrition-related risk spectrum during incretin-based therapy

### 3.1 Reduced food intake and dietary pattern shifts

Appetite suppression and smaller meals are expected pharmacologic effects of incretin-based therapy; in obesity-related HFpEF, the clinical concern is whether the reduced intake still supplies adequate protein, micronutrients, and fluid while maintaining stability (59). In a randomized 6-week phase 1 trial, tirzepatide reduced *ad libitum* energy intake, hunger, food cravings, and responsiveness to food cues, demonstrating measurable changes in ingestive behavior beyond deliberate dietary restraint (60). Oral semaglutide produced a similar pattern in adults with obesity, lowering energy intake and appetite while improving control of eating, which positions reduced intake as a class-relevant nutritional issue (61). Tirzepatide also changed participant-reported cravings and food preferences, suggesting that treatment may alter both the quantity and the composition of food selected (62).

Real-world observations extend these trial findings, linking incretin-based therapy to changes in taste perception and appetite regulation among people with overweight or obesity (63). Food purchase data after glucagon-like peptide-1 receptor agonist (GLP-1RA) initiation show lower energy density and reduced sugar, carbohydrate, and saturated fat purchases, although purchases remain an imperfect proxy for actual intake and nutritional adequacy (64). Cross-sectional dietary data from adults with T2D receiving GLP-1RAs reveal potential deficits in diet quality that may be relevant to metabolically complex HFpEF, while remaining outside an HFpEF-specific evidence base (65). Clinical practice has outpaced nutrition infrastructure, with drug-assisted weight loss reshaping appetite and food choice more rapidly than structured dietary guidance has entered routine prescribing (66). Once gastrointestinal intolerance is superimposed, the intended reduction in intake can progress to inadequate protein, micronutrient, and fluid consumption (67).

### 3.2 Gastrointestinal adverse events and inadequate protein and micronutrient intake

Gastrointestinal symptoms during incretin-based therapy carry nutritional as well as tolerability consequences, with delayed gastric emptying, altered gut motility, and intensified satiety narrowing food choice, reducing meal size, and complicating hydration in patients whose HFpEF care depends on stable volume status (68). In pooled semaglutide 2.4 mg trials, nausea, diarrhea, vomiting, and constipation were more frequent than with placebo, clustered during dose escalation, and caused discontinuation in a minority of participants (69). In SURMOUNT-5, tirzepatide produced greater weight reduction than semaglutide, while gastrointestinal adverse events remained common with both agents; monitoring of tolerance and intake remains necessary irrespective of potency (70). A network meta-analysis in nondiabetic adults with overweight or obesity likewise identified nausea, vomiting, diarrhea, and constipation as the predominant gastrointestinal events across GLP-1RA regimens, with risks varying by agent and dose (71). Placebo-controlled randomized evidence further defines the boundary: GLP-1RAs increased the risks of cholelithiasis and gastroesophageal reflux disease, without a uniform excess across all gastrointestinal or biliary disorders (72).

Reported intake among GLP-1RA users brings these consequences into sharper focus, with shortfalls in fiber, calcium, iron, magnesium, potassium, choline, and vitamins A, C, D, and E and protein intake per kilogram often below levels commonly targeted during weight loss (73). A separate analysis found that low consumption of fruits, vegetables, whole grains, seafood, and plant proteins may persist during treatment, locating nutrient inadequacy in the dietary pattern itself and not solely in supplement use (74). A pilot study in T2D also raises the possibility that semaglutide reduces intestinal iron absorption, placing iron risk at the intersection of intake and absorption (75). For HFpEF, this is clinically relevant because malnutrition is associated with worse short-term outcomes, making persistent inadequate intake more than an innocuous accompaniment to weight loss (76). High-protein dietary interventions may improve nutritional, muscle-related, and functional outcomes in HF, although HFpEF-specific evidence for dose, distribution, and tolerability remains limited (77). Taken together, gastrointestinal intolerance and nutrient inadequacy link early changes in eating to later concerns over bone, muscle compartments, adherence, and treatment interruption (78).

### 3.3 Bone mass, muscle compartments, adherence, and post-discontinuation rebound

Semaglutide can sustain substantial weight loss for 104 weeks, shifting safety assessment toward the long-term preservation of bone, muscle compartments, adherence, and maintenance (79). In adults at increased fracture risk, once-weekly semaglutide did not increase bone formation as measured by procollagen type I N-terminal propeptide and was accompanied by higher bone resorption and lower areal bone mineral density, supporting skeletal surveillance during active weight reduction (80). Exercise may attenuate this concern: in a secondary analysis of a randomized trial, the combination of exercise and GLP-1RA therapy better preserved bone health than GLP-1RA therapy alone while supporting weight loss (81). Matched real-world cohort data in patients at increased fracture risk provide an additional caution and support baseline and follow-up bone assessment in susceptible groups (82). Muscle-related change is equally nuanced, as SURPASS-3 MRI associated tirzepatide with lower muscle volume alongside potentially favorable reductions in muscle fat infiltration in people with T2D (83). Body composition substudies of newer polyagonists such as retatrutide reinforce the same requirement: as pharmacologic weight loss increases, fat and nonfat compartments should be measured and reported directly (84).

Adherence ultimately links early efficacy to durable outcomes, yet real-world cohorts show frequent GLP-1RA discontinuation among patients treated for obesity, T2D, or both (85). Among adults in the United States receiving dual-labeled GLP-1RAs for overweight or obesity, discontinuation within the first year was common and reinitiation was less frequent in those without T2D (86). SURMOUNT-4 illustrates the physiological consequence of withdrawal, with continued tirzepatide maintaining or augmenting weight reduction and placebo substitution leading to substantial regain (87). A systematic review and meta-analysis found a similar metabolic rebound after GLP-1RA cessation, including deterioration in body weight and cardiometabolic markers (88). In obesity-related HFpEF, long-term nutrition support serves more than relapse prevention, as weight cycling may worsen body composition and increase vulnerability to sarcopenia or sarcopenic obesity in patients who are already functionally limited (89).

### 3.4 Identifying patients at high nutritional risk

Identifying nutritional risk extends beyond formal contraindications, particularly in older adults with obesity, in whom potential weight-loss benefits may be offset by sarcopenia, bone loss, frailty, or functional decline (90). STEP-HFpEF DM nevertheless shows that semaglutide improves symptoms, physical limitations, and body weight in obesity-related HFpEF with T2D, establishing this metabolically complex subgroup as an active treatment population (15). Real-world comparative data in cardiometabolic HFpEF also show that recipients of semaglutide or tirzepatide often carry complex comorbidity profiles beyond those represented by trial eligibility criteria, limiting the usefulness of randomized controlled trial (RCT) eligibility criteria alone for nutritional screening (91). Sarcopenic obesity is a particularly important phenotype at this intersection, defined by excess adiposity together with low muscle mass or low muscle function and directly relevant when incretin-based therapy is initiated in HFpEF (92).

Closer monitoring is warranted for patients predisposed to sarcopenia by older age, low baseline muscle reserve, CKD, liver disease, inflammatory disorders, or low activity during GLP-1RA-associated weight loss (93). Body composition studies in obesity-related HFpEF support this approach, as BMI cannot capture appendicular lean mass, skeletal muscle quality, or cardiorespiratory fitness (94). Malnutrition introduces a separate layer of vulnerability; even in early-stage HFpEF, it has been associated with exercise intolerance, impaired metabolic and hemodynamic responses, and worse outcomes (95). Particular caution is needed in older adults, for whom evidence on pharmacotherapy is expanding more quickly than evidence on preserving bone, muscle, and daily function in frail or multimorbid states (96). In patients with reduced intake or other clinical features suggesting nutrient deficiency, laboratory assessment may include hemoglobin, iron indices such as ferritin and transferrin saturation, serum calcium, folate, and vitamin B12, guided by the individual clinical context (20, 67). Table 2 situates each risk signal within the population and treatment context in which it was observed and links it to a corresponding MNT response. In patients vulnerable to sarcopenic obesity, physical function, muscle and bone health, gastrointestinal tolerance, and the expected benefits for obesity-related complications should be considered together when incretin-based therapy is initiated or continued (97).

**Table 2:** Evidence on nutrition-related risks during incretin-based therapy and practical clinical response cues.

Evidence class/source	Population/context	Incretin-based exposure	Key risk signal	MNT response cue	References
Phase 1 RCT	Adults with overweight or obesity, no diabetes	Tirzepatide vs. placebo	*Ad libitum* lunch intake reduced by approximately 525 kcal; appetite and cravings reduced	Early intake review; protein-prioritized small meals	(60)
Cross-sectional study	69 adults using a GLP-1RA for ≥1 month	GLP-1RA use	Low fiber, calcium, iron, magnesium, potassium, choline, and vitamins A, C, D, and E; protein intake (g/kg) below levels commonly targeted during weight loss	Diet record, protein target, and micronutrient screening	(73)
Real-world cohort/research letter	Danish receipt-donor cohort	GLP-1RA initiation	Purchases shifted toward lower energy density, sugar, carbohydrates, and saturated fat	Review actual foods, affordability, and diet quality	(64)
Pooled safety analysis	Adults with overweight or obesity	Semaglutide 2.4 mg	Nausea 43.9%, diarrhea 29.7%, vomiting 24.5%, constipation 24.2%; GI discontinuation 4.3%	Titration support; low-fat small meals; hydration and constipation plan	(69)
Phase 3b, open-label RCT; SURMOUNT-5	Adults with obesity, no T2D	Tirzepatide vs. semaglutide	Greater weight and waist reduction with tirzepatide; GI events common during escalation	Intake, hydration, and GI monitoring during titration	(70)
DXA substudy of SURMOUNT-1	Adults with obesity or overweight; *n* = 160	Tirzepatide	Weight −21.3%; fat mass −33.9%; lean mass −10.9%; ~25% of weight loss from lean mass	Track weight, waist, and function; consider DXA or BIA	(44)
Prospective clinical study; SEMALEAN	Patients with obesity	Semaglutide 2.4 mg	Fat mass decreased; lean mass fell early then stabilized; handgrip improved	Combine body composition testing with grip or walk testing	(45)
Phase 2 RCT	Adults with increased fracture risk	Semaglutide 1.0 mg vs. placebo	CTX increased; lumbar spine and total hip aBMD were lower; weight decreased	Bone-risk review; protein, calcium, vitamin D, and resistance exercise	(80)
Secondary analysis of RCT	Adults after diet-induced weight loss	Exercise, liraglutide, combination, or placebo	Exercise-containing strategies favored BMD preservation during larger weight loss	Resistance and weight-bearing exercise with adequate protein, calcium, and vitamin D	(81)
Randomized withdrawal RCT; SURMOUNT-4	Adults with obesity or overweight, no diabetes	Continued tirzepatide vs. withdrawal	Withdrawal caused regain; 89.5 vs. 16.6% maintained ≥80% of prior loss	Maintenance nutrition and activity plan before stopping	(87)
Off-treatment extension	Adults after semaglutide 2.4 mg trial	Semaglutide withdrawal	1 year after withdrawal, about two-thirds of lost weight was regained	Post-cessation nutrition and activity planning	(156)
Real-world retrospective cohort	125,474 adults in the United States with overweight or obesity	Dual-labeled GLP-1RA initiation	1-year discontinuation: 64.8% without T2D, 46.5% with T2D; GI adverse events linked to stopping	Access, adherence, GI, and reinitiation monitoring	(86)

Most evidence derives from populations with obesity, type 2 diabetes, or other high-risk conditions; HFpEF-specific data are limited. The MNT response cues are practical considerations inferred from these data and have not been prospectively evaluated as an integrated strategy in HFpEF. aBMD, areal bone mineral density; BIA, bioelectrical impedance analysis; BMD, bone mineral density; CTX, C-terminal telopeptide; DXA, dual-energy X-ray absorptiometry; GI, gastrointestinal; GLP-1RA, glucagon-like peptide-1 receptor agonist; HFpEF, heart failure with preserved ejection fraction; MNT, medical nutrition therapy; RCT, randomized controlled trial; T2D, type 2 diabetes.

## 4 Lean mass preservation, muscle function, and symptom improvement in HFpEF

### 4.1 Do body composition changes and functional benefits move together?

The central question in obesity-related HFpEF is whether changes in fat mass, lean compartments, muscle quality, cardiac loading, and physical performance evolve together during incretin-based therapy (98). Outside HFpEF, the SURMOUNT-1 DXA substudy documented reductions in body weight, fat mass, and lean mass with tirzepatide, making direct measurement of weight-loss composition essential (44). SEMALEAN adds an important qualification: semaglutide 2.4 mg reduced fat mass, produced an early decline in lean mass that later stabilized, and improved handgrip strength, so body composition cannot stand in for muscle function (45). In older patients with obesity and HFpEF, long-term follow-up after caloric restriction and aerobic training found parallel improvements in body composition and exercise capacity, although the gains diminished over time (99). HuMAIN-HFpEF provides a contrasting example, as a fat-directed metabolic accelerator improved body weight and adiposity-related composition without a significant parallel increase in peak VO_2_, leaving composition–function synchrony as an empirical question (100).

Functional benefit requires direct measurement, as illustrated in SUMMIT, where tirzepatide improved health status, quality of life, NYHA functional class, 6MWD, global symptom impression, and medication burden—domains that cannot be reconstructed from changes in fat or lean mass (57). A STEP-HFpEF analysis by sex further challenges a weight-centric reading: women lost more weight than men, yet improvements in HF symptoms, physical limitations, and exercise function were broadly similar (101). Analyses of NYHA functional class in the STEP-HFpEF program likewise support functional class as an endpoint in its own right, independent of any assumed downstream relation to body weight (102). Echocardiographic data add a cardiac dimension, with semaglutide-related symptom improvement occurring alongside favorable changes in cardiac structure and function as well as peripheral body composition change (103). HF-specific assessment of muscle health also requires individualization, since age- and sex-specific cutoffs improve prognostic classification in older patients with HF (104). Body composition is indispensable to the evaluation of weight-loss quality, but it remains incomplete without measures of strength, walking performance, symptoms, and HFpEF-specific health status (105).

### 4.2 Skeletal muscle metabolism and exercise intolerance in HFpEF

Beyond its contractile role, skeletal muscle contributes to glucose disposal, lipid oxidation, amino acid turnover, and exercise-induced endocrine signaling (106). In patients with HFpEF, reduced skeletal muscle perfusion reserve is associated with exercise intolerance independently of ejection fraction and central filling pressures (107). These observations support direct assessment of peripheral muscle function when symptoms persist despite apparently stable cardiac measures.

Experimental studies offer plausible explanations for this peripheral limitation. In a mouse model of cardiometabolic HFpEF, voluntary exercise restored skeletal muscle fatty acid and branched-chain amino acid oxidation and improved exercise capacity (108). In a rat model, short-term caloric restriction corrected an impaired muscle growth response after resistance exercise (109). Musclin also improved oxidative capacity and exercise tolerance in a murine HFpEF model (110). Broader work on exerkines describes signaling between skeletal muscle and cardiovascular, metabolic, immune, and neurological tissues (111). Its clinical relevance in HFpEF remains to be established.

Human intervention data remain limited. The OptimEx biopsy substudy identified exercise-modality-dependent changes in molecular and mitochondrial markers, some of which diminished after detraining (112). In a 12-month randomized trial, combined endurance and resistance training did not improve the primary composite endpoint; peak VO_2_ and NYHA functional class improved (113). Ferric Carboxymaltose and Exercise Capacity in Heart Failure With Preserved Ejection Fraction and Iron Deficiency (FAIR-HFpEF) showed that correction of iron deficiency can influence 6MWD, linking a nutrition-related abnormality with a functional endpoint in HFpEF (114). Current evidence implicates skeletal muscle dysfunction in exercise intolerance. The evidence base consists mainly of preclinical studies, small human substudies, and trials of exercise or iron repletion; direct trials of MNT during incretin-based therapy are lacking (115). Protein adequacy and exercise exposure should therefore be considered together, with direct measurement of strength, walking capacity, symptoms, and health status (116).

### 4.3 High-protein intake, protein distribution, resistance training, and multimodal lifestyle intervention

Diet and physical activity intervention remain integral when pharmacologic appetite suppression is used in obesity-related HFpEF, and the magnitude of drug-induced weight loss heightens the need for deliberate lifestyle support (117). Incretin-based obesity medications create a predictable nutritional challenge, as treatment-assisted weight loss can compromise adequacy unless dietary support begins early (118). A protein prescription is meaningful only within the wider context of usual intake, diet quality, fiber, micronutrients, hydration, gastrointestinal tolerability, renal function, and access to protein-rich foods (119). Source and timing also matter: heterogeneous protein sources and meal distribution patterns among community-dwelling older adults show why total daily intake can miss clinically relevant gaps (120).

Higher protein intake during weight-loss interventions in older adults has been associated with selected lean soft tissue outcomes, but inconsistent results preclude treating protein alone as a functional intervention (121). Across adults, meta-analytic evidence is more consistent when protein is paired with resistance exercise, which supports lean mass and strength through the combination of nutritional substrate and mechanical stimulus (122). In older patients with obesity and HFpEF, adding resistance training to caloric restriction plus aerobic exercise increased leg strength, demonstrating that the exercise prescription can be directed toward muscle preservation even without evidence for hard HF outcomes (123). Outside HFpEF, liraglutide combined with exercise reduced metabolic syndrome severity, abdominal obesity, and inflammation, offering a plausible drug–exercise synergy that still requires HFpEF-specific testing (124). The same combination has skeletal implications: during weight-loss maintenance, exercise added to GLP-1RA therapy preserved bone health better than GLP-1RA therapy alone (81).

Lifestyle intervention should be organized as a structured co-therapy with incretin-based therapy, using diet quality, physical activity, and behavioral support to shape tolerability, durability, and the composition of weight loss (125). Within this structure, MNT can convert changes in appetite and limited intake into a coordinated nutrition–activity plan that protects protein adequacy and body composition without reducing care to a supplement recommendation (126). This coordination is particularly important in older patients and those with low muscle reserve, given the prevalence of sarcopenia and sarcopenic obesity and their association with all-cause mortality (127). During substantial weight loss, assessment should include muscle strength, weight-bearing activity, and physical performance alongside body weight to determine whether functional benefit is being achieved in HFpEF (128).

### 4.4 Is there a risk that greater weight loss does not translate into greater functional benefit?

Percentage weight loss remains useful for trial reporting, yet it cannot define the therapeutic target in obesity-related HFpEF because it does not capture mobility, muscle function, symptoms, or clinical vulnerability (129). In the prespecified STEP-HFpEF analysis across obesity classes and achieved weight reduction, semaglutide improved symptoms, physical limitations, exercise function, inflammation, and body weight, with larger weight losses accompanying greater clinical benefit; weight therefore remains relevant (31). Inflammation analyses nonetheless show that C-reactive protein reduction and functional improvement cannot be compressed into a simple linear consequence of weight loss (130). N-terminal pro-B-type natriuretic peptide findings provide a parallel caution, as semaglutide lowered the biomarker while improving symptoms and functional capacity through mechanisms that extend beyond mechanical unloading alone (131). STEP-HFpEF DM analyses across baseline hemoglobin A1c levels further show that metabolic phenotype must be specified when interpreting the relationships among weight loss, symptoms, and exercise function (132).

**Figure 2:**
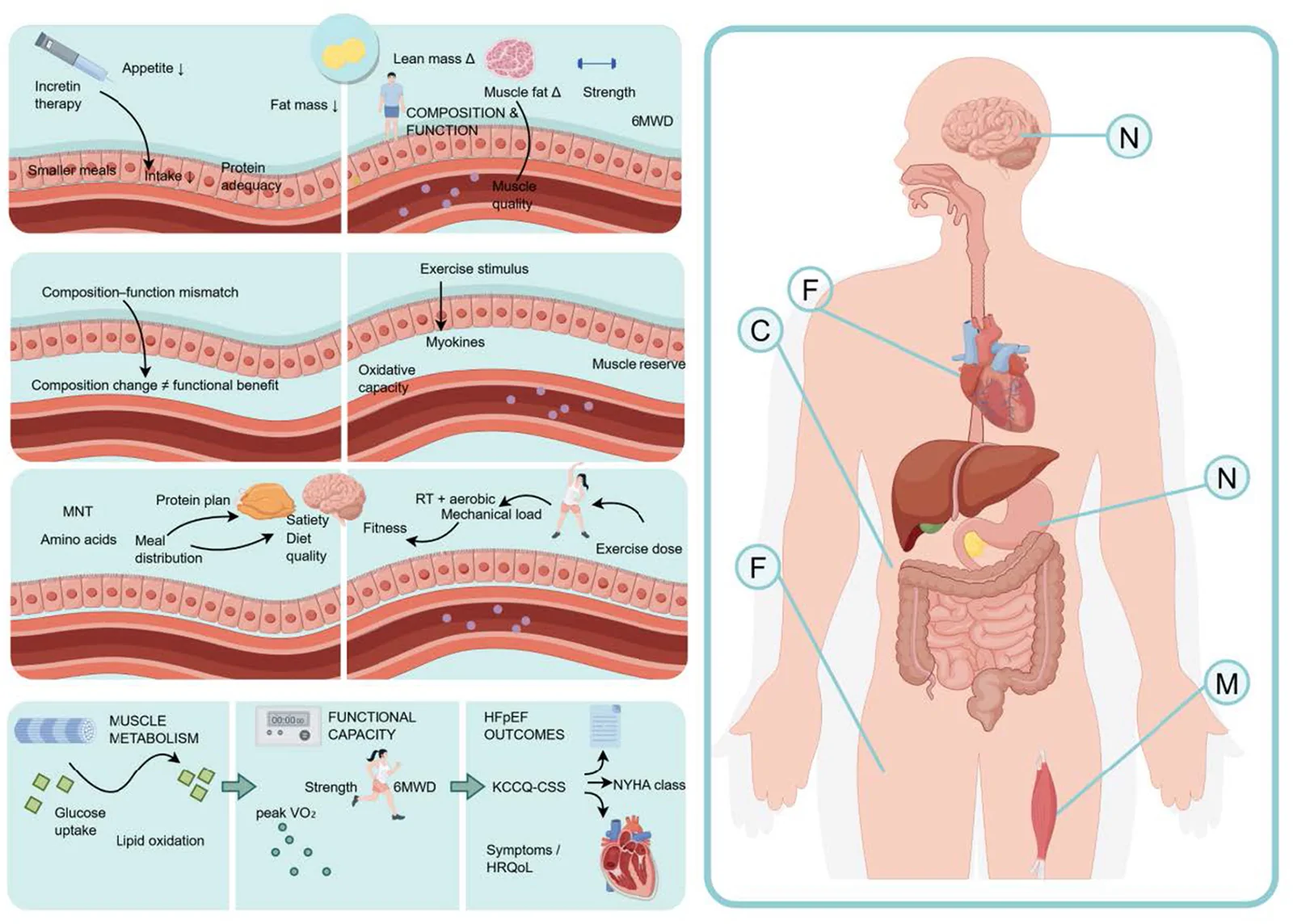
Relationships among body composition, skeletal muscle metabolism, nutrition–exercise co-therapy, and functional outcomes during incretin-based therapy in obesity-related HFpEF. The N, C, M, and F callouts denote nutrition and intake regulation, body composition and adiposity, skeletal muscle, and functional capacity or HFpEF outcomes, respectively. The left panels summarize changes in appetite and food intake, body composition, exercise exposure, and muscle metabolism. Body-composition changes are shown separately from strength and walking performance because these measures may not change in parallel. MNT includes amino acid and protein planning, meal distribution, satiety-aware eating, and diet quality, coordinated with resistance and aerobic exercise. The lower sequence links muscle glucose uptake and lipid oxidation with strength, 6MWD, peak VO_2_, KCCQ-CSS, NYHA functional class, symptoms, and HRQoL. Arrows denote proposed biological or clinical associations and do not establish causality. Tissue and circulation elements are schematic. The figure was created using Figdraw. 6MWD, 6-min walk distance; C, body composition and adiposity; F, functional capacity and HFpEF outcomes; HFpEF, heart failure with preserved ejection fraction; HRQoL, health-related quality of life; KCCQ-CSS, Kansas City Cardiomyopathy Questionnaire Clinical Summary Score; M, skeletal muscle; MNT, medical nutrition therapy; N, nutrition and intake regulation; NYHA, New York Heart Association; peak VO_2_, peak oxygen uptake; RT, resistance training; Δ, change without a prespecified direction; ↓, decrease.

Comorbidity also changes the meaning of weight loss, as the high prevalence of atrial fibrillation in the STEP-HFpEF program makes rhythm phenotype and baseline functional reserve relevant to interpretation of treatment response (133). Muscle data impose a second boundary: current translational and proof-of-concept clinical evidence has not established that GLP-1RA-associated weight loss causes disproportionate loss of muscle mass or function, and lean mass change alone remains insufficient for judgment (134). Broader evidence on cardiorespiratory fitness supports direct measurement, as fitness carries risk information that BMI does not capture (135). Accordingly, assessment in obesity-related HFpEF should include body weight, adiposity, dietary intake, body composition, muscle performance, symptoms, and HF status, consistent with AOM trials that pair weight reduction with complication-oriented safety outcomes (136). Figure 2 summarizes the relationships among these measures and the roles of MNT and exercise during incretin-based therapy.

## 5 Medical nutrition therapy pathway for clinical care

### 5.1 Assessment before treatment initiation

Before incretin-based therapy begins, pretreatment assessment should establish obesity as an HF-specific therapeutic phenotype by linking adiposity with symptoms, cardiorenal risk, treatment goals, and monitoring targets (4). Clinicians should first confirm and stabilize HFpEF, addressing congestion, blood pressure, atrial fibrillation, CKD, T2D, OSA, and background diuretic therapy so that dyspnea and edema are not attributed to body size without adequate evaluation (2). Cardiovascular weight-management guidance then informs drug selection, contraindication screening, optimization of comorbidities, team responsibilities, and follow-up cadence before escalation (137). SUMMIT data on effect modification by BMI, central adiposity, and changes in weight or waist support documenting BMI, waist circumference, waist-to-height ratio, recent weight trajectory, and patient-prioritized functional goals at baseline (32).

Nutrition assessment should proceed in parallel with the cardiometabolic evaluation, drawing on dietary history, malnutrition risk, sodium and fluid context, and energy and protein adequacy to interpret symptoms, treatment tolerance, and recovery potential in HF (138). A pretreatment diet record is especially useful before appetite suppression begins, given reported shortfalls in protein, fiber, calcium, iron, magnesium, potassium, choline, and vitamins A, C, D, and E among GLP-1RA users (73). The same visit should screen for body-composition risk, as sarcopenic obesity—excess adiposity combined with low muscle mass or function—can be missed by BMI (92). Outpatient HF screening tools identify overlapping yet nonidentical risk groups; selection should reflect setting, age, frailty burden, and capacity for follow-up instead of relying on a single universal score (139). Baseline iron status and function are also needed, as FAIR-HFpEF links treatment of iron deficiency to exercise capacity endpoints, so fatigue, 6MWD, or another functional measure is required to interpret subsequent symptom change (114).

### 5.2 Nutritional strategies during dose escalation

Dose escalation is a phase of nutritional adaptation in which next-generation incretin therapies can rapidly alter appetite, GI tolerance, meal size, and the required cadence of clinical review (140). Anticipatory counseling before each dose increase helps patients distinguish expected satiety from clinically important inadequate intake and report nausea, vomiting, constipation, or reduced fluid and protein intake before treatment stability is compromised (141). In pooled semaglutide 2.4 mg trials, nausea, diarrhea, vomiting, and constipation clustered during escalation and caused discontinuation in only a minority, favoring early dietary adjustment over passive observation (69). Real-world practice adds another concern: GLP-1RA-based weight-loss therapies are often used at lower doses than in trials, while first-year discontinuation remains substantial and may be driven in part by gastrointestinal symptoms (142). Persistent gastrointestinal symptoms or a marked reduction in dietary variety should also prompt review of micronutrient adequacy. When food-based measures are insufficient, supplementation should be guided by dietary assessment, clinical findings, and laboratory results (20).

In obesity-related HFpEF, titration occurs against background congestion risk and diuretic exposure; semaglutide improved symptoms and physical limitations across diuretic subgroups and was accompanied by lower loop-diuretic use and dose over follow-up (143). Secondary SUMMIT analyses extend the HF-specific rationale for surveillance, showing that tirzepatide reduced circulatory volume–pressure overload, systemic inflammation, and cardiovascular–kidney end-organ injury (144). These favorable cardiorenal findings do not justify permissive dehydration; low intake, vomiting, diarrhea, diuretic therapy, CKD, or symptomatic hypotension should trigger review of fluid intake, blood pressure, renal function, and congestion (145). SURMOUNT-3 also keeps lifestyle care in view, as tirzepatide followed an intensive lifestyle intervention and was not tested as a substitute for structured dietary and activity support (146). Routines established during titration—symptom-responsive meal adjustment, protein prioritization in small meals, HF-tailored hydration and renal monitoring, and behavioral support—should continue into plateau and maintenance care (147).

### 5.3 Maintenance and plateau-phase management

Maintenance shifts the clinical objective from dose-driven weight reduction to chronic obesity care, with pharmacotherapy, nutrition, physical activity, and behavioral support adjusted to complications, treatment burden, and patient-defined functional goals (148). A plateau is a physiological phase, not evidence of personal failure: models of dietary restriction, glucagon-like peptide-1 receptor agonism, and bariatric surgery attribute weight stabilization to adaptive changes in energy expenditure, appetite feedback, and treatment exposure (149). Tirzepatide data also argue against judging response from a single early weight measurement, as later responders in SURMOUNT-1 could still achieve clinically meaningful loss by week 72 with continued treatment (150). When weight loss slows, clinicians should reassess adiposity-related complications, dietary adequacy, treatment tolerance, function, and patient priorities before intensifying therapy solely to restart scale change (151).

Plateau assessment in obesity-related HFpEF must retain a functional focus, as resistance training added to caloric restriction plus aerobic exercise improved strength-related outcomes in older patients with obesity and HFpEF, although the trial was not designed for hard HF events (123). A larger HFpEF trial of combined endurance and resistance training missed its primary composite endpoint yet improved peak VO_2_ and NYHA functional class, supporting direct measurement of exercise capacity and symptoms during plateau care (113). Body composition should be monitored alongside function, because DXA-based placebo-controlled evidence suggests that a meaningful proportion of GLP-1RA-associated weight loss is lean mass and available bone mineral density data remain insufficient to assume skeletal neutrality (152). Patient-reported outcomes provide a further non-scale target: in SURMOUNT-1, tirzepatide-associated weight reduction correlated with better HRQoL in adults with obesity, although the evidence is not HFpEF-specific (153). Shared decision-making can then determine whether a plateau represents satisfactory stabilization, a need to reinforce nutrition and activity, a reason to intensify functional monitoring, or a transition toward dose reduction or discontinuation planning (154).

### 5.4 Dose reduction, discontinuation, and long-term weight maintenance

Dose reduction or discontinuation should mark entry into a planned MNT maintenance phase, with diet quality, protein-prioritized meals, resistance and aerobic activity, symptom surveillance, and follow-up frequency agreed before drug exposure changes (20). Such planning is clinically necessary: among adults in the United States with overweight or obesity receiving dual-labeled GLP-1RAs, first-year discontinuation was common and reinitiation remained incomplete, especially among those without T2D (86). Implementation factors frequently determine persistence, including cost, insurance coverage, availability, adverse effects, and perceived effectiveness of semaglutide or tirzepatide (155). The STEP 1 extension illustrates the biological consequences of stopping semaglutide, with substantial weight regain and partial loss of cardiometabolic improvements after withdrawal of semaglutide 2.4 mg and lifestyle intervention (156). SURMOUNT-4 yielded the same directional message for tirzepatide: continued treatment maintained or augmented weight reduction, whereas placebo substitution led to marked regain (87).

**Figure 3:**
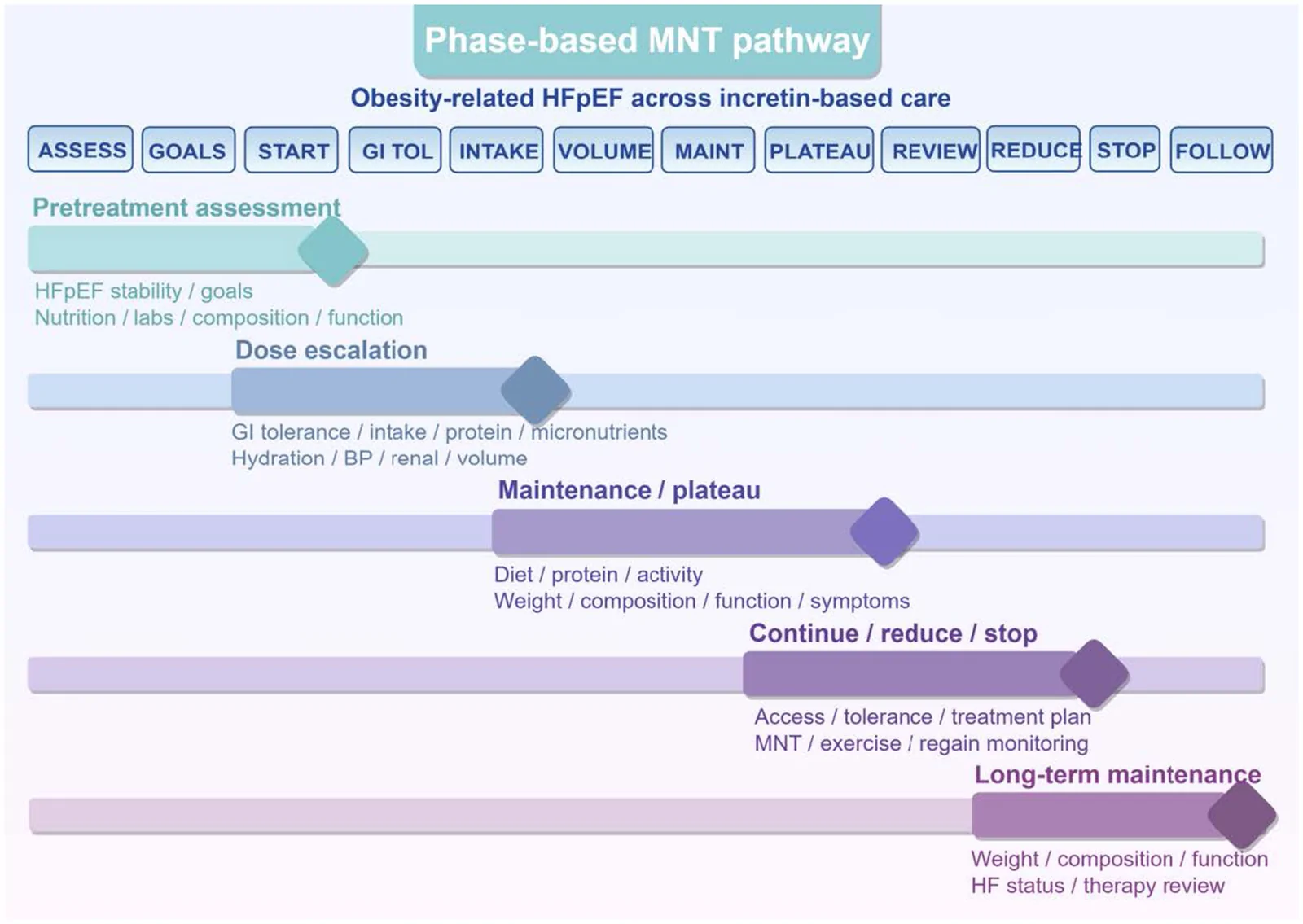
Proposed phase-based medical nutrition therapy pathway for incretin-treated obesity-related HFpEF. The sequence follows common clinical decision points in HFpEF care and obesity pharmacotherapy: pretreatment assessment, dose escalation, maintenance or plateau, dose reduction or discontinuation, and long-term follow-up. Pretreatment assessment covers HFpEF stability, patient-prioritized goals, nutritional status, selected laboratory measures, body composition, and functional capacity. During dose escalation, monitoring focuses on gastrointestinal tolerance, food and protein intake, micronutrient adequacy, hydration, blood pressure, renal function, and volume status. Micronutrient supplementation is considered when dietary assessment, clinical findings, or laboratory results indicate a need. Maintenance and plateau care include reassessment of diet and protein adequacy, physical activity, weight and body composition, function, symptoms, and treatment goals before any attempt to intensify therapy solely to restart weight loss. Subsequent treatment decisions may include continuation, dose reduction, or discontinuation according to access, tolerability, treatment burden, clinical response, and shared decision-making. MNT, resistance and aerobic activity, and monitoring for weight regain should be established before medication exposure is reduced or stopped. Long-term maintenance continues surveillance of weight, body composition, function, HF status, and the need to continue, adjust, reinitiate, or change obesity therapy. The horizontal positions and bar lengths are conceptual and do not represent fixed or proportional treatment durations. Stages may overlap, recur, or be omitted according to clinical status. REDUCE and STOP are conditional options rather than mandatory consecutive steps. Diamonds indicate reassessment or treatment-decision points; darker bar segments indicate the period of greatest stage-specific emphasis, whereas lighter extensions indicate ongoing longitudinal surveillance. This pathway synthesizes clinical guidance and evidence for individual components; it has not been tested as an integrated intervention in a randomized trial of incretin-treated obesity-related HFpEF. The figure was created using Figdraw. BP, blood pressure; GI, gastrointestinal; GI TOL, gastrointestinal tolerance; HF, heart failure; HFpEF, heart failure with preserved ejection fraction; MAINT, maintenance; MNT, medical nutrition therapy.

Rebound after treatment cessation belongs within chronic-disease management, as a systematic review and meta-analysis found rapid weight regain and reversal of cardiometabolic benefits after weight-management medications were stopped, although most evidence came from populations outside HFpEF (157). For some patients, dose reduction may be more feasible than abrupt cessation; in SURMOUNT-MAINTAIN, continued tirzepatide, including a 5-mg maintenance dose, sustained weight reduction more effectively than placebo withdrawal (158). Nutrition and exercise support should be in place before tapering starts, given randomized post-treatment evidence that prior supervised exercise combined with GLP-1RA therapy preserved weight loss and body composition better after cessation than GLP-1RA therapy alone (159). Long-term follow-up also requires flexibility, as real-world data show frequent reinitiation or use of alternative obesity treatment after semaglutide or tirzepatide discontinuation and wide variation in subsequent weight change; Table 3 translates these observations into a phase-based MNT pathway linking medication status with nutrition, activity, body composition, HF symptoms, and re-escalation planning (160) (Figure 3).

**Table 3:** Evidence informing a proposed phase-based MNT pathway for obesity-related HFpEF during incretin-based therapy.

Evidence class/source	Care phase	Population/context	Pathway component	Pathway role/key message	References
Joint advisory from professional societies	Whole pathway	Adults receiving incretin-based therapy for obesity	Nutrition and lifestyle support	Diet screening, GI management, prevention of nutrient deficiencies, muscle and bone protection, RDN support, and post-discontinuation maintenance	(20)
ACC Scientific Statement	HF-specific obesity care	Adults with obesity and HF	HF-specific obesity assessment	Diagnosis, risk assessment, lifestyle therapy, AOM therapy, surgery, monitoring, and evidence gaps	(4)
ACC expert consensus/concise guidance	Medical weight management initiation	Adults requiring weight management for cardiovascular health	Cardiometabolic weight management framework	Diagnosis, medication selection, comorbidity optimization, and team-based care	(137)
ACC Expert Consensus Decision Pathway	Baseline HFpEF assessment	Patients with HFpEF	HFpEF diagnosis and longitudinal care	Diagnostic, comorbidity, nonpharmacologic, and team-based HFpEF pathway	(2)
ESPEN/EASO consensus statement	Pretreatment screening	Individuals at risk of sarcopenic obesity	Sarcopenic obesity definition and diagnosis	Excess adiposity plus low muscle mass or function; staged diagnostic approach	(92)
Phase 3 RCT; SURMOUNT-3	Lifestyle-to-drug intensification	Adults with overweight or obesity after ≥5% lifestyle-induced weight loss	Tirzepatide after intensive lifestyle intervention	Substantial additional weight loss after lifestyle lead-in; GI adverse events common	(146)
RCT	Active weight loss and muscle function protection	Older adults with obesity-related HFpEF	CR + AT ± RT	RT improved leg strength and muscle quality; both arms improved peak VO_2_ and KCCQ	(123)
RCT	Functional recovery/rehabilitation	Patients with HFpEF	Endurance + resistance training	Primary endpoint neutral; peak VO_2_ and NYHA functional class improved	(113)
RCT	Drug–exercise integration	Adults with obesity after low-calorie diet	Exercise, liraglutide, combination, or placebo	Combination reduced abdominal fat and hsCRP more than placebo	(124)
Post-treatment analysis of RCT	Discontinuation and maintenance	Adults with obesity after diet-induced weight loss	Exercise, liraglutide, or combination; 1-year off-treatment follow-up	Exercise plus liraglutide better maintained weight and fat loss than liraglutide alone	(159)
RCT; FAIR-HFpEF	HF-specific laboratory monitoring	Symptomatic HFpEF with iron deficiency	Ferric carboxymaltose vs. placebo	6MWD improved by 49 m at 24 weeks; secondary endpoints not significant	(114)

The proposed pathway draws on clinical guidance and evidence for its individual components. No trial has evaluated incretin-based therapy, structured MNT, and cardiac rehabilitation as an integrated intervention in obesity-related HFpEF. 6MWD, 6-min walk distance; ACC, American College of Cardiology; AOM, anti-obesity medication; AT, aerobic training; CR, caloric restriction; EASO, European Association for the Study of Obesity; ESPEN, European Society for Clinical Nutrition and Metabolism; GI, gastrointestinal; HF, heart failure; HFpEF, heart failure with preserved ejection fraction; hsCRP, high-sensitivity C-reactive protein; KCCQ, Kansas City Cardiomyopathy Questionnaire; MNT, medical nutrition therapy; NYHA, New York Heart Association; peak VO_2_, peak oxygen uptake; RCT, randomized controlled trial; RDN, registered dietitian nutritionist; RT, resistance training.

## 6 Evidence gaps and future agenda

### 6.1 Evidence gap in randomized trials of MNT in HFpEF

HFpEF lifestyle trials show that structured exercise and broader lifestyle programs can improve exercise capacity and HRQoL, yet none directly tests whether protocolized MNT makes incretin-induced weight loss more nutritionally secure or function-preserving (161). Evidence for dietary intervention is narrower and highly heterogeneous in intervention type, intensity, comparator, and outcome selection, leaving no trial-tested MNT prescription for obesity-related HFpEF (162). Pragmatic weight-management programs suggest that meaningful weight loss and functional improvement are achievable in real-world HFpEF care, but they cannot isolate the contributions of contemporary nutrition support, protein adequacy, body-composition monitoring, or rehabilitation in the era of incretin-based therapy (163). Broader HF nutrition trials define the boundary: in SODIUM-HF, a single sodium-restriction target did not reduce cardiovascular hospitalization, emergency-department visits, or all-cause death, showing why one dietary rule cannot substitute for phenotype-specific MNT evaluation (164).

This gap has greater consequence in the era of incretin-based therapy, given the preexisting vulnerability of many patients with HFpEF to malnutrition and sarcopenia and the absence of prospective trials testing whether structured nutritional protection preserves muscle function during pharmacologic weight loss (165). Supervised exercise training has a stronger functional evidence base in HFpEF, but no obesity-related HFpEF RCT has systematically combined it with incretin-based therapy, MNT, adherence support, and access strategies (166). Future randomized programs could use cardiac rehabilitation infrastructure to integrate baseline assessment, nutrition counseling, weight and body composition surveillance, aerobic and resistance training, functional endpoints, and evaluation of program quality (167).

### 6.2 Nutritional and body composition endpoints for future incretin trials

Semaglutide trials in obesity-related HFpEF already include patient-centered and functional endpoints—Kansas City Cardiomyopathy Questionnaire Clinical Summary Score (KCCQ-CSS), 6MWD, body weight, and inflammatory markers—that future studies should retain while adding prospective nutrition and body composition measures (19). SUMMIT establishes a complementary hierarchy for tirzepatide: worsening HF or cardiovascular death, health status, walking distance, and body weight remain essential, as nutritional surrogates cannot by themselves demonstrate HFpEF benefit (16). Prospective nutrition phenotyping should capture usual intake, protein and micronutrient adequacy, appetite-limited intake, barriers to assimilation, weight-loss trajectory, risk of low muscle mass, and inflammatory or disease burden (168). Body composition assessment should extend beyond BMI to fat mass, lean soft tissue, appendicular lean mass, regional adiposity, and visceral or ectopic fat, using methods selected for precision, feasibility, and longitudinal comparability (169).

Muscle endpoints require the same precision: DXA-derived lean mass cannot represent skeletal muscle health without complementary measures of muscle quantity, quality, strength, and physical performance (170). When incretin-based therapy is paired with exercise or cardiac rehabilitation, trials should also measure cardiorespiratory fitness, chair-stand performance, relative strength, daily activity, and exercise adherence, while clearly identifying which supporting evidence is extrapolated from obesity populations outside HFpEF (171). KCCQ domains, symptom burden, physical limitation, and patient-perceived function should remain primary patient-relevant outcomes, not secondary annotations of weight or body composition (172). A coherent endpoint hierarchy would connect nutrition adequacy with body composition, muscle function, HFpEF symptoms, functional capacity, and HF events, allowing the intervention to be evaluated as integrated nutrition–pharmacotherapy–rehabilitation rather than a weight-loss trial with ancillary nutrition measures (173).

### 6.3 From “weight loss medication plus generic diet advice” to an integrated pathway

The next step is to replace generic dietary advice with a reproducible cardiovascular weight-management pathway that can be evaluated across HF and preventive cardiology settings, while embedding nutrition variables prospectively in incretin trials (137). Such a pathway treats obesity in cardiovascular disease as a complication-oriented cardiometabolic condition and coordinates nutrition, physical activity, pharmacotherapy, risk-factor control, and longitudinal surveillance across symptomatic, metabolic, and volume-sensitive domains (174). Current incretin studies rarely standardize nutrition support, dietitian involvement, or protein and micronutrient monitoring sufficiently to determine whether weight loss is high quality or comparable between trials (175). MNT should be prespecified as an evidence-based dietary program with behavioral support, dietary pattern selection, monitoring, clinically indicated co-interventions, explicit targets, and reassessment triggers; a brief instruction to eat less does not meet that standard (176).

Care delivered by a registered dietitian nutritionist offers an implementation model by individualizing energy targets, protein and micronutrient adequacy, GI tolerance adjustments, food access, and maintenance planning across treatment phases (177). Physical activity and resistance-oriented rehabilitation should accompany pharmacotherapy to protect fitness, strength, physical function, and muscle quality during active weight loss and maintenance, establishing exercise as a functional co-therapy (178). When food insecurity, cost, or adherence barriers constrain participation, integrated pathways can incorporate food-is-medicine or nutrition security components and evaluate diet quality, food security, retention, function, and clinical outcomes as implementation endpoints; adherence then becomes a measure of pathway performance (179).

## 7 Discussion

Randomized trials and recent meta-analyses show that incretin-based therapy improves health status, walking capacity, and HF-related outcomes in obesity-related HFpEF, together with substantial weight reduction (180). Clinical interpretation should therefore include HF events, KCCQ-CSS, 6MWD, physical limitation, symptom burden, and durability of response (181).

The composition and functional consequences of treatment-associated weight loss remain less certain. Scale weight and DXA-derived lean mass do not distinguish changes in fat, fluid, skeletal muscle, or muscle quality, and their clinical meaning depends on direct measures of strength and physical performance (182). Nutritional intake, gastrointestinal tolerance, exercise exposure, and body composition have rarely been assessed together in incretin trials, leaving the contribution of structured MNT unresolved (183).

The proposed MNT pathway brings these clinical tasks together across treatment initiation, dose escalation, maintenance, and discontinuation. The synthesis is narrative and does not include quantitative pooling or formal risk-of-bias assessment. Its recommendations draw on HFpEF trials, obesity care, nutrition studies, and exercise research; no randomized trial has tested the complete approach in incretin-treated obesity-related HFpEF. Future studies should prespecify dietary intake, micronutrient status, body composition, strength, walking capacity, symptoms, health status, HF events, and treatment persistence.

In clinical practice, the goal is durable improvement in symptoms, physical function, nutritional status, quality of life, and HF stability during and after pharmacologic weight loss. Whether structured MNT improves the safety, functional value, and persistence of these benefits requires direct testing.
