# Correction: Medical nutrition therapy in incretin-treated obesity-related heart failure with preserved ejection fraction

**DOI:** 10.3389/fnut.2026.1970493

**Published:** 2026-09-01

**Authors:** Qiuli Ming, Ze Li

**Affiliations:** 1 The Third Affiliated Hospital of Henan Medical University, Xinxiang, China; 2 The First Affiliated Hospital and College of Clinical Medicine, Henan University of Science and Technology, Luoyang, China

**Keywords:** body composition, HFpEF, incretin-based therapy, medical nutrition therapy, muscle function, obesity

Author, Jiankun Cui, was erroneously included as an author and corresponding author in the published article. The correct author list reads:

Qiuli Ming^1*^ and Ze Li^2^

^1^The Third Affiliated Hospital of Henan Medical University, Xinxiang, China

^2^The First Affiliated Hospital and College of Clinical Medicine, Henan University of Science and Technology, Luoyang, China

The correct corresponding author is Qiuli Ming.

The **Author Contributions** statement has been corrected to read: “QM: Conceptualization, Writing – review & editing, Writing – original draft, Visualization. ZL: Writing – original draft, Writing – review & editing, Validation.”

The incorrect **Funding Statement** was erroneously given as, “The author(s) declared that financial support was received for this work and/or its publication. This work was supported by the Natural Science Foundation of Heilongjiang Province (Grant No. PL2025H250).” The correct **Funding Statement** is, “The author(s) declared that financial support was not received for this work and/or its publication.”

The original version of this article has been updated.

